# Supercritical Fluid CO_2_ Extraction of Essential Oil from Spearmint Leaves Dried by Vacuum Drying with a Desiccant

**DOI:** 10.3390/foods15020213

**Published:** 2026-01-07

**Authors:** Rustam Tokpayev, Zair Ibraimov, Khavaza Tamina, Bauyrzhan Bukenov, Bagashar Zhaksybay, Amina Abdullanova, Yekaterina Chshendrygina, Kanagat Kishibayev, Luca Fiori

**Affiliations:** 1 Center of Physical and Chemical Methods of Research and Analysis, al-Farabi Kazakh National University, 96A, Tole bi, Almaty 050012, Kazakhstan; rustamtokpaev@mail.ru (R.T.); k.tamina@mail.ru (K.T.); bukenov.b.o@gmail.com (B.B.); bagazhaksybay@gmail.com (B.Z.); abdullanova0300@mail.ru (A.A.); kateshennik@gmail.com (Y.C.); kanagat_kishibaev@mail.ru (K.K.); 2 Department of Civil Environmental and Mechanical Engineering, University of Trento, via Mesiano 77, 38123 Trento, Italy; luca.fiori@unitn.it

**Keywords:** spearmint, vacuum drying, desiccant, supercritical CO_2_ extraction, essential oil, carvone

## Abstract

The essential oil (EO) of *Mentha spicata* L. (spearmint) exhibits pronounced biological activity, making it valuable for applications in agrochemistry as an insecticidal agent, in perfumery and cosmetics, and as a natural preservative in the food industry. However, maintaining the integrity and yield of EO during post-harvest processing and extraction remains a key technological challenge. This study aimed to enhance the vacuum-drying (VD) process of spearmint using calcium chloride as a desiccant and to optimize the conditions of supercritical CO_2_ extraction (SC-CO_2_), including EO separation and the evaluation of its solubility under dynamic extraction conditions. The incorporation of calcium chloride into the VD process reduced drying duration by 21.1% and processing costs by 31.0%, while increasing EO yield by 11%. A decrease in separator pressure from 70 to 10 bar during SC-CO_2_ extraction resulted in nearly a threefold increase in EO yield by minimizing the loss of volatile constituents. The solubility of spearmint EO in supercritical CO_2_ was successfully described by the Chrastil model and correlated with carvone solubility. The maximum total phenolic content (72.3 ± 2.2 mg gallic acid equivalent per gram) was observed at a CO_2_ density of 353.91 kg/m^3^. The solubility of EO was studied directly using the plant matrix under dynamic conditions.

## 1. Introduction

Nowadays, the scientific community worldwide has a high level of interest in plant-based raw materials and their processing products, e.g., essential oils (EOs), as they are alternatives for synthetic products, the use of which causes negative effects [1,2]. EOs are oily liquids, with typical smell and colors varying from light yellow to dark green, possessing biological activity [3]. In European countries, the EO consumption market was estimated at USD 11.7 billion in 2023, and its predicted growth will be 8.4% between 2024 and 2030 [4].

Spearmint (*Mentha spicata* L.) is a herbaceous plant from the *Lamiaceae* family; its aboveground part is highly aromatic. It is known that spearmint types with menthol-containing EO are grown in India on over 200 thousand hectares of fields, and annual EO production is approximately 25–28 thousand tons [5]. Due to high biological activity and a rich chemical composition, spearmint EO has practical application in multiple areas [6]: it is used in medicine for coughs, body aches, and stress. It is a culinary component in sauces, drinks and confectionery products, as well as a component in perfumery and cosmetics [7].

Spearmint EO includes mono- and sesquiterpenes, phenylpropanoids, and compound esters, as well as nitrogen- and sulfur-containing compounds. Primary spearmint EO compounds are α-pinene, 1,8-cineole (eucalyptole), linalool, menthone, isomenthone, neomenthol, menthol, pulegone, carvone, etc. [8]. Moreover, according to research by a Flavor Extract Manufacturers Association (FEMA) expert group in 2015, it is recognized that primary compounds of spearmint EO are safe for humans [9].

Traditionally, spearmint EO extraction starts with raw material sample preparation. As a rule, the EO content in the plant’s aboveground part is the highest in the early blossom period. Then, raw material undergoes sorting and drying; then EOs are extracted from dried raw material through various methods [10].

Drying is a necessary step before EO extraction for several reasons: the storage life of fresh raw material is short; EO extraction from fresh raw material can be incomplete; the density of fresh raw material is relatively high, which leads to a high liquid–solid (L:S) ratio during EO extraction; ground raw material is prone to biochemical reactions, which causes EO quality deterioration; EO extraction from fresh raw material using the SC-CO_2_ method causes ice formation in the system, which in its turn causes pressure [11].

Drying significantly reduces the moisture content of the raw material, which not only simplifies EO extraction processes but also helps prevent blockages and abnormal pressure increases that may occur during extraction, particularly in high-pressure systems [12]. The following drying methods are currently known: natural (in the shade or under direct sunlight), convectional, lyophilic, microwave, microwave in vacuum, infrared and SC-CO_2_ drying [13]. One of the key moments in drying is the loss of volatile EO compounds due to evaporation and thermal decomposition [14]. Vacuum drying is the most optimal. It is characterized by plants’ low shrinkage ratio, relatively low duration, and low loss of desirable components [15]. One of the approaches allowing a minimization of the process duration is use of air desiccants such as calcium chloride, sodium sulphate, etc. [16].

Extraction is the main way of obtaining EO. Traditional extraction methods such as hydro- and steam distillation, organic solvent extraction (Soxhlet method), maceration, percolating extraction, liquid extraction under pressure and many others have significant disadvantages: they require long periods of time, have a risk of fire and explosion, are toxic, need high temperatures, and require solvent removal [17]. CO_2_ in supercritical state has unique properties: gas-like viscosity, higher diffusion coefficient and solubility than in organic solvents, availability, fire and explosion safety, and low toxicity [11].

Currently, there are no studies on SC-CO_2_EO extraction from plant-based materials dried through vacuum drying with a desiccant [18]. There are studies on separating b-carotene from lutein through SC-CO_2_ extraction from spearmint leaves dried using lyophilization [19]; there is research on the SC-CO_2_ extraction of biologically active flavonoids from spearmint leaves dried using convective methods [20]. Moreover, optimizing conditions in separators during dynamic SC-CO_2_ extraction is also extremely insufficiently studied. Due to the fact that spearmint EO components include highly volatile thermolabile compounds, special attention must be paid to these compounds’ recovery in separators. There are already studies on the fractionation of EO components, e.g., EO extraction from basil and marjoram using a system of two separators with various pressures [21].

In order to estimate EO solubility, researchers often use previously obtained EOs. This approach is not suitable for describing actual SC-CO_2_ extraction from plant-based materials. During extraction from plant-based materials the process is influenced by plant cell structure resistance, which is not considered while describing processes of EO dissolving in SC-CO_2_.

This study’s objective is modification of vacuum drying using calcium chloride as a desiccant and researching processes of extraction and separation as well as spearmint EO’s solubility in SC-CO_2_ under dynamic conditions. The results of vacuum drying with a desiccant—improvements in extraction and separation conditions—enabled the production of peppermint essential oil with a high yield. Our investigation of essential oil solubility in supercritical CO_2_ under dynamic conditions, which correlated with carvone solubility, broadens the understanding of its solubility under real conditions.

## 2. Materials and Methods

### 2.1. Initial Raw Materials and Reagents

This study used spearmint *M. spicata* L., also known as spearmint, purchased at a grocery market Altyn Orda, Almaty, Kazakhstan, grown at plantations of Almaty region, Kazakhstan. Twigs were separated from weeds; healthy leaves were separated from stems and dry or rotten leaves. For experiments on moisture and mineral compounds, the following were used: toluene (CAS: 108-88-3, ACS reagent, ≥99.5%, Sigma-Aldrich, St. Louis, MO, USA) and hydrochloric acid (CAS: 7647-01-0, chemically pure according to GOST 3118-77, JSC EKOS-1, Saint Petersburg, Russia). For drying, anhydrous calcium chloride was used (CAS:10043-52-4, pure, JSC Lenreaktiv). For performing supercritical CO_2_ extraction, carbon dioxide was used (top grade, 99.8%, LLP Ikhsan Technogas, Almaty, Kazakhstan); for dissolving obtained EOs, dichloromethane was used (CAS:75-09-2, puriss. p.a., ACS reagent, reag. ISO, ≥99.9% (GC), Sigma-Aldrich). Carvone (CAS: 6485-40-1, 97% purity) and the internal standard camphor (CAS: 76-22-2, 96% purity) were purchased from Meryer (Shanghai Meryer Chemical Group Co., Ltd., Shanghai, China) for the preparation of calibration solutions.

### 2.2. Moisture and Ash Content Determination

Moisture determination was carried out through Dean–Stark method according to ISO 939:2021 [22]. For this, an aliquot of dried or fresh spearmint leaves of approximately ~10.00–100.00 g was put into a flask of 250 mL volume. Then, 50–100 mL toluene was added to the leaves, so that they were completely submerged in it. Toluene was driven off with water. According to the amount of off-driven water, moisture was determined.

Moisture was calculated using Equation (1):

(1) $$w=\frac{100\times V}{m},$$

where V—the volume of water collected in the Dean–Stark trap (mL); m—mass of spearmint leaves used to determine moisture (g).

Ash content was determined through calcination of dried spearmint leaves in a SNOL 7.2/1100L muffle furnace (UAB SNOL, Narkunai, Lithuania) at 200 °C for 1 h, then at 550 °C for 10 h [23].

Ash content was calculated using Equation (2):

(2) $$A=\frac{100\times m1}{m2},$$

where m1—mass of spearmint leaves after calcination (g); m2—mass of spearmint leaves before calcination (g).

### 2.3. Drying

The study involved researching vacuum drying with and without a desiccant, convective drying at 30, 40, and 50 °C, and natural drying in the shade at room temperature (22 °C). Vacuum drying was carried out in an AKTAN VTSh-K52-250 vacuum heating chamber (LLP Aktan Vakuum, Fryazino, Russia); convective drying was carried out in a SNOL 58/350 laboratory drying chamber (SnolTherm, UAB, Narkunai, Lithuania).

In order to study the kinetics of vacuum, convective and natural shade drying, spearmint leaves weighed to an amount of 5.0 g were placed in a thin layer onto pans and dried until reaching equilibrium moisture.

The vacuum-drying chamber contained two aluminum shelves, each of which held 150.0 g of the plant material (spearmint leaves). When the desiccant was used, 300.0 g of calcium chloride was placed in a separate plastic container positioned at the center of both the upper and lower shelves. This configuration prevented any direct contact between the desiccant and the plant material and eliminated the risk of sample contamination. Data obtained through experimental spearmint leaf drying were tested using various mathematical models. For choosing the optimal model for describing the drying process, the following statistical parameters were calculated: coefficient of determination (R^2^), sum squared error (SSE) and root-mean-square error (RMSE). The closer R^2^ is to 1 and the smaller SSE and RMSE, the better a model describes the drying process. Relative moisture or moisture ratio (MR) was defined as the ratio between water or moisture content (MC_t_) at any moment of time to initial moisture (MC_0_), and was determined according to Equation (3) [24]:

(3) $$MR=\frac{{MC}_{t}}{{MC}_{0}},$$

### 2.4. Calculation of Vacuum-Drying Cost with and Without Desiccant

The cost of vacuum-drying was calculated to compare drying with and without a desiccant. The cost depends on drying duration, the dryer’s capacity and the set tariff per 1 kWh. According to the data of the Alatau Zharyk Companyasy—EnergoSbyt Joint Stock Company, Almaty, Kazakhstan (https://esalmaty.kz/ru/business-tariffs, accessed on 1 December 2025), power cost in the Republic of Kazakhstan, namely in the city of Almaty, for installations funded by the state budget (III group) since 1 December 2025 is KZT 42.45 or USD 0.08 (according to Republic of Kazakhstan National Bank rates of 1 December 2025).

Vacuum drying cost with and without desiccant was calculated using Equation (4):

(4) Vacuum drying cost=power capacity×duration×tariff per 1 kWh,

It should be noted that the cost of vacuum drying with desiccant includes desiccant restoration.

### 2.5. EO Content Determination

Determination of EO content in fresh and dried spearmint leaves was performed using Clevenger hydrodistillation method according to GOST ISO 6571-2016 [25]: 200 g of fresh leaves or 40 g of dried leaves was placed into a 1 L round-bottomed flask with 600 mL water, and EO was distilled with water for 4 h. The volume of obtained EO was determined using a graded scale trap.

EO content in 100 g of leaves was calculated using Equation (5):

(5) $$EO\;content=\frac{V\times 100}{m},$$

where V—spearmint EO volume (mL); m—spearmint leaves’ mass (g).

### 2.6. Mineral Compound Content Determination in Dried Spearmint Leaves

Mineral compounds in dried spearmint leaves were determined according to [23]. After ash content determination, obtained ash was dissolved in 5 mL of 20% hydrochloric acid solution. The resulting solution was analyzed for metal content using a Shimadzu АА-6200 atomic absorption spectrometer (Kyoto, Japan).

Metal content (mg/kg) was calculated using Equation (6):

(6) $$Metal\;content=\frac{C\times V}{m},$$

where C—metal concentration in obtained solution after leaf mineralization (mg/L); V—analyzed solution volume (L); m—spearmint leaves’ mass (kg).

### 2.7. Supercritical Fluid CO_2_ Extraction

A study of SC-CO_2_ extraction processes was carried out on a laboratory unit (Figure S1). Herein, 2.5 g of dried and ground leaves (≤0.7 mm size) was placed into an extractor (Ex) with a 100 mL volume pre-heated to 40 ± 0.1 °C. CO_2_ liquid from the tank went through an SFT-10 pump and filtered elements into the extractor until reaching the required pressure with a set flow rate (12 mL/min). Pressure in the extractor and separator was controlled using regulators R1 and R2. Upon reaching the required pressure, R1 was opened and R2 maintained the necessary pressure in the separator. A study of the pressure’s impact in separator S1 was carried out within the range of 10 to 70 bar. The duration of the dynamic extraction stage, starting from the opening of valve R1, was 120 min. Then the pressure in the separator and extractor was reduced and EO mass was measured. In order to collect EO at all stages, a pre-weighed vial of 15.0 mL volume was placed into separator S1. EO mass was determined based on the difference in vial mass. Obtained EO in the vial was dissolved in 3.0 mL of dichloromethane for further qualitative and quantitative composition analysis. EO was temporarily stored in a Biryusa 290 refrigerator chamber (JSC Biryusa, Krasnoyarsk, Russia) at 2 °C until its composition was determined. After selection of the optimal pressure value in separator S1 for maximum EO yield, the CO_2_ flow rate was optimized from 1.0 to 5.0 mL/min.

### 2.8. Study of EO Solubility

EO solubility was investigated at an optimal CO_2_ flow rate and at different CO_2_ densities (353.91, 602.58, 736.1, 830.09, 900.3 kg/m^3^). Solubility of EO was studied in dynamic mode directly from dried spearmint leaves. Previous studies have primarily focused on the solubility of pure oils, using glass beads as an inert matrix [26,27] or paper towers impregnated with oil [28]. In contrast, the present study evaluates EO solubility directly from plant material, which is of greater practical relevance. The experimental conditions and operating parameters were similar to those used in the extraction steps described above.

EO solubility was defined as the ratio of EO collected in the separator to the mass of CO_2_ that passed through the extractor at a given flow rate and density.

The Chrastil mathematical model based on the dependence of CO_2_ density and solubility was used to describe EO solubility [29]:

(7) $$lnS=a_{0}+a_{1}ln\rho +\frac{a_{2}}{T},$$

where lnS—spearmint EO solubility (mg/g); а_0_, а_1_, а_2_—Chrastil model constants; ρ—CO_2_ density (kg/m^3^); Т—temperature (K).

Model constants were determined using the Solution Search tool in Microsoft Excel software 2021 (Microsoft, Redmond, WA, USA).

### 2.9. Qualitative and Quantitative Analysis of Spearmint EO Using Gas Chromatography–Mass Spectrometry (GC-MS)

GC-MS analyses were performed using an Agilent 6890N gas chromatograph with a 5973N mass spectrometer with a diffusion pump (Agilent Technologies, Santa Clara, CA, USA), equipped with a split/splitless inlet and a Combi-PAL autosampler (CTC Analytics, Zwingen, Switzerland). Herein, 1 µL aliquot of each calibration and sample solution was injected into the GC inlet at 240 °C in split mode with a split ratio of 200:1. Separation was carried out on a non-polar DB-5MS UI capillary column (30 m × 0.25 mm i.d., 0.25 µm film thickness; J&W, Agilent Technologies, Santa Clara, CA, USA) with helium (>99.995%, Orenburg-Tehgas, Yuzhny Ural village, Russia) as the carrier gas at a constant flow rate of 1.0 mL/min. The oven temperature was programmed from 40 °C (held for 5 min) to 250 °C at a heating rate of 10 °C/min. The total GC run time was 26 min. The ion source, quadrupole, and interface temperatures were set at 230, 150 and 310 °C, respectively. Detection was performed using electron ionization (EI) at 70 eV in SCAN mode over the *m/z* range of 10–550. Data acquisition and analysis were carried out using MSD ChemStation software version E.02.02.1431.

Calibration solutions of carvone were prepared in the concentration range of 0.1–5.0 mg/mL. Camphor was used as internal standard and added to each calibration and sample extract solution to achieve a final concentration of 1 mg/mL.

### 2.10. Total Phenol Content (TPC) Determination in Spearmint EO Obtained at Different CO_2_ Densities

TPC determination was performed using the method described by [16] with minor changes. For this, an ethanol solution of spearmint EO and Folin–Ciocalteu reagent (20 μL each) were placed into cells with 2 mL bidistilled water. The mixture was left in a dark place for 30 min. Then saturated sodium carbonate solution (50 μL) was added and the mixture was left for 30 more min. After the reaction was complete, optical density was measured at a wavelength of 725 nm. The TPC was determined using gallic acid to obtain calibration curves. Results were presented as mg equivalent of gallic acid/g (mg EGA/g).

### 2.11. Statistical Analysis

All experiments were performed in triplicate (*n* = 3). The results were expressed as mean ± confidence interval. Statistical analysis was carried out using Microsoft Excel. The mean, standard deviation, and 95% confidence intervals were calculated assuming a normal distribution and a confidence level of 95%. The final values presented in the tables and graphs represent the average of three independent measurements with their corresponding confidence intervals.

## 3. Results and Discussion

### 3.1. Moisture and Ash Content 

The moisture content of spearmint leaves was determined using the Dean–Stark method and found to be 78.3 ± 1.5%. Control of the moisture content in dried raw material is essential, as it can significantly affect the SC-CO_2_ extraction process. For example, the authors of [30] reported that the extract yield from *Helichrysum italicum* flowers in SC-CO_2_ extraction was higher at 28.4% moisture compared to 10.5%. The ash content of spearmint leaves in this study was 12.57 ± 0.03%, which is consistent with previous findings of 10.86% reported in [31].

### 3.2. Determination of Macro- and Micronutrient Content in Spearmint Leaves

Heavy metals (HMs) are toxic and potentially harmful to the environment, as they may be present in air, water, and soil. Many plant species can accumulate HMs in roots, leaves, and stems, and thus can be used in remediation processes for cleaning contaminated soils [32]. In addition, aromatic herbs such as spearmint, citronella, basil, lemongrass, palmarosa, and vetiver may accumulate various HMs, acting as natural phytoremediators. Essential elements such as zinc, iron, calcium, and potassium are important for plant and human nutrition, and aromatic herbs are generally rich sources of these micronutrients [33].

In the dried spearmint leaves analyzed in this study, the total macronutrient content was 63,480 ± 810 mg/kg, while micronutrients totaled 780 ± 40 mg/kg (Table S1). Specifically, the contents of potassium, manganese, copper, lead, and cadmium were approximately twice as high, zinc and sodium approximately four times higher, and calcium approximately twice lower compared to previously reported values for peppermint (*Mentha x piperita* L.) [34]. Moreover, the metal content in the plant material was compared with the maximum permissible concentrations (MPCs) for soils, showing that manganese, iron, copper, nickel, zinc, and lead concentrations did not exceed MPCs [35].

### 3.3. Effect of Drying on the Samples

The dependence of the MR of spearmint leaves on process time (t) can be represented by drying curves. Increasing the drying temperature reduced the process time. The optimal vacuum-drying temperature was 40 °C, at which the drying time was approximately 1.5 times shorter than at 30 °C (Figure S2a). The convective drying rate was 2–4 times higher compared to that of vacuum drying (Figure S2b). This behavior can be explained by the circulation of hot air within the drying chamber, which continuously removes the released moisture. A similar dependence has been reported for the convective and vacuum drying of mushrooms and parsley [36]. Under natural drying conditions, approximately ~24 h was required for the moisture ratio to reach its equilibrium value corresponding to the initial raw material (Figure S2c). It can be noted that shade drying of *Mentha spicata* L. at room temperature (25 ± 2 °C) required 28 h [37].

During vacuum drying, water vapor condenses on the colder door of the drying chamber. This condensation increases the moisture level inside the chamber, thereby reducing the drying rate [38].

Modification of drying methods often involves the combination of various physical processes, such as ultrasonic treatment, microwave heating, and infrared radiation. For example, the application of ultrasonic-assisted microwave vacuum drying was shown to enhance both the drying efficiency and quality of dried mushrooms [39]. Preliminary osmodehydration followed by pulsed vacuum drying of tomato pieces under infrared radiation resulted in products with well-developed microchannels and porous structures [40]. In another study, vacuum freeze-drying of kiwi slices pretreated with ultrasound yielded a final moisture content of 4.85% [41].

The use of calcium chloride as an air desiccant has been well established in numerous studies and is now widely applied in both laboratory and industrial settings [42,43]. The incorporation of a desiccant reduces the amount of condensed moisture in the drying system, which positively affects the drying time. For example, liquid lithium chloride is commonly employed in air-conditioning and dehumidification systems [44]. In [45], the performance of conventional desiccants (calcium chloride, lithium chloride, and potassium formate) was compared with that of innovative ionic liquids. It was shown that the moisture sorption capacity of CaCl_2_ at 25 °C is 0.10 g H_2_O per g of desiccant, whereas the ionic liquid [EMIM][OAc] exhibits a significantly higher capacity of 0.42 g H_2_O per g. However, unlike CaCl_2_, ionic liquids cannot be effectively regenerated. Calcium chloride can be regenerated by desorption of up to 0.30 g H_2_O per g of desiccant. In [46], the influence of CaCl_2_ on tomato drying in an indirectly forced-convection solar dryer was investigated, and the use of CaCl_2_ reduced the moisture content of tomatoes from 1270.27 to 1142.87 g. Furthermore, the study reported in [47] demonstrated that a cast membrane containing 3.13% CaCl_2_ provided a 70% higher air-dehumidification efficiency compared with a membrane containing 13.04% CaCl_2_ under vacuum conditions (80 kPa).

**Figure 1:**
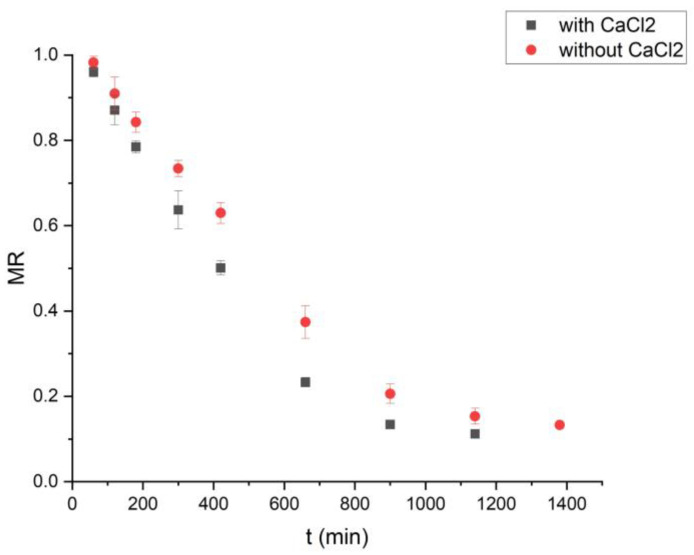
Effect of vacuum drying time with/without desiccant on spearmint leaves’ moisture ratio (m = 150.0 g at 40 °C).

Vacuum drying with a desiccant at 40 °C proceeded 21.1% faster than vacuum drying without a desiccant (Figure 1). Increasing the vacuum-drying temperature above 40 °C is impractical, as the moisture-adsorption capacity of the desiccant decreases significantly [16]. Conversely, vacuum drying with a desiccant at temperatures below 40 °C required longer processing times, exceeding 12 h.

### 3.4. Cost of Vacuum Drying with/Without Desiccant

The total cost of vacuum drying with a desiccant for 1 kg of fresh spearmint leaves was KZT 1400.19 (USD 2.73), of which KZT 1188.01 (USD 2.32) was attributed to the drying process and KZT 212.19 (USD 0.41) to desiccant regeneration. In contrast, the cost of vacuum drying without a desiccant for 1 kg of fresh spearmint leaves was KZT 1771.39 (USD 3.46). Thus, vacuum drying with a desiccant was 26.5% less expensive than vacuum drying without a desiccant.

### 3.5. Drying Process Modeling

The drying processes of spearmint leaves with and without a desiccant were described using 12 mathematical models based on a linearized relationship, ln(MR) = f(t) (Tables S2 and S3). The equations include the moisture ratio (MR), drying time (t), model constants (k, k_0_, k_1_, g, h), and drying coefficients (a, b, c, n). Among the evaluated models, the modified trinomial Henderson and Pabis model provided the best fit for vacuum drying both with and without CaCl_2_ as a desiccant. The coefficients of determination (R^2^) were 0.9887 and 0.9882, respectively.

This model is widely applied to describe drying processes. For example, it effectively describes the convective drying of spearmint at 50 °C, with a coefficient of determination (R^2^) of 0.9981. In addition, this model has been successfully used to describe the drying of meat and ginger root slices [48,49].

### 3.6. EO Content 

The EO content in dried spearmint leaves is expressed as milliliters of EO per 100 g of dried raw material (Table 1). The highest EO content was observed for spearmint leaves dried by vacuum drying at 30 °C, reaching 1.33%. EO content in leaves dried through natural drying in the shade reached 1.10%, and that in fresh leaves reached 0.62%. The EO content in leaves dried naturally in the shade was 1.10%, whereas fresh leaves contained 0.62% EO. Vacuum drying with a desiccant resulted in an EO content that was 7.8% higher than that obtained without a desiccant. However, the EO content of spearmint leaves dried by vacuum drying with a desiccant was 8.2% lower than that of leaves dried by convective drying at the same temperature.

Similar findings were reported in [50], where *Mentha pulegium* L. was dried under natural shade, direct sunlight, and thermal conditions. The EO content of leaves dried under sunlight was 1.4%, while thermal drying at 50 °C resulted in an EO content of 1.1%, and shade drying yielded the highest EO content (1.8%). In contrast, the study reported in [51] demonstrated an opposite trend: the EO content of *Mentha aquatica* L. leaves dried by thermal treatment at 50 °C (1.47%) was higher than that of leaves dried naturally (0.81%).

### 3.7. Qualitative and Quantitative Composition of Spearmint EO

GC–MS analysis revealed that the primary compounds identified in spearmint leaves were carvone, limonene, and eucalyptol (1,8-cineole) (Table S4). These results are consistent with previous studies [52,53], which report carvone as the dominant constituent of *Mentha spicata* L. essential oil.

The concentration of limonene was nearly twice as high in spearmint leaves dried by vacuum drying with a desiccant at 40 °C compared with leaves dried by vacuum drying without a desiccant. The highest eucalyptol concentration was also observed in spearmint leaves dried by vacuum drying with a desiccant at 40 °C. In contrast, the maximum carvone concentration was detected in fresh spearmint leaves (77.61%). Carvone concentrations in leaves dried by vacuum drying with and without a desiccant at 40 °C were approximately 10% higher than those in leaves dried naturally or by convective drying, and were comparable to the values obtained for convective drying at 50 °C.

### 3.8. Effect of Supercritical CO_2_ Extraction on the Samples

#### 3.8.1. Study of Separator Pressure Impact on Efficiency of EO Separation from CO_2_

Current studies on fractionation processes are mainly based on the use of two or more separators operated under different conditions [54,55,56], or on single-separator systems in which fractionation is achieved through dynamic extraction under varying conditions [57,58]. However, the influence of separator pressure remains insufficiently investigated.

As the pressure increased from 10.0 to 70.0 bar, the total EO yield decreased from 1.52 ± 0.05% to 0.12 ± 0.06%. According to the National Institute of Standards and Technology (NIST), within the temperature range of −20 to 25 °C and at a pressure of 70 bar, CO_2_ exhibits properties close to those of liquid CO_2_, in which essential oils can remain dissolved. Under these conditions, EO separation from CO_2_ becomes inefficient, and part of the EO is carried away with the CO_2_ stream.

**Figure 2:**
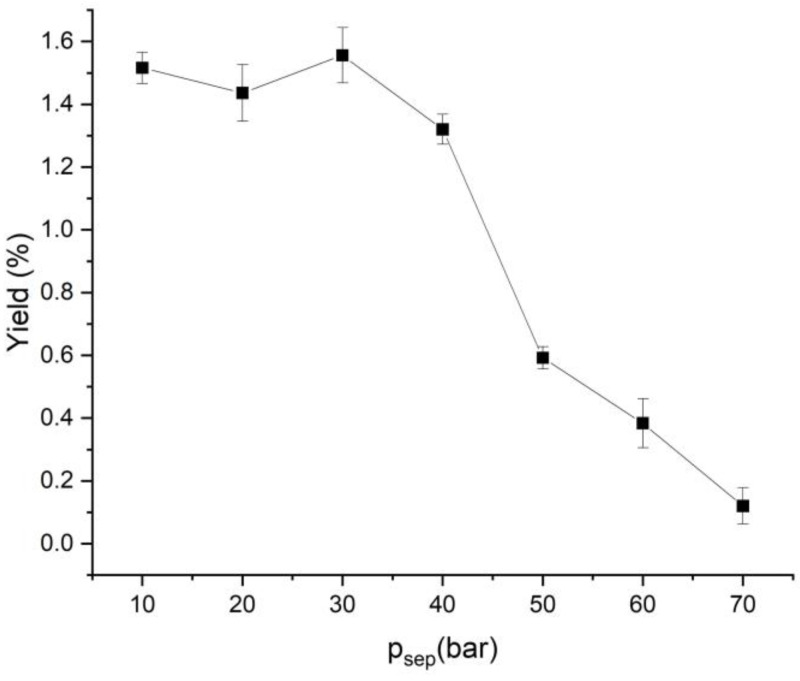
Effect of separator pressure on spearmint EO yield.

When the separator pressure increased from 10 to 30 bar, the spearmint EO yield showed a slight increase; however, a further rise in pressure led to a significant decline in yield. At elevated pressures during dynamic extraction, EO components can be entrained and removed from the separator by the CO_2_ flow. Moreover, at a separator pressure of 70 bar, CO_2_ exhibits a substantially higher solvent power than at 10 bar, which further reduces EO separation efficiency (Figure 2).

The pressure difference between the separator and the atmosphere at 70 bar is significantly greater than at 10 bar, which leads to the occurrence of a throttling effect at the outlet orifice of the separator tubing. Using the gas-flow-through-an-orifice equation (Equation (8)), the CO_2_ mass flow rate can be calculated [59]:

(8) $$m=C_{d}A_{0}\rho \surd (2RT_{sep})\surd (\frac{k}{k-1}[{\left(P_{r}/P_{s}\right)}^{\frac{2}{k}}-{\left(P_{r}/P_{s}\right)}^{\frac{k}{k+1}}]),$$

where m—CO_2_ mass flow rate (kg/s); C_d_—coefficient of discharge through orifices; A_0—_orifice cross section (m^2^); ρ—CO_2_ density (kg/m^3^); R—universal gas constant (J/(kg × K)); T_sep_—separator temperature (K); k—gas combination index; P_r_—atmospheric pressure (bar); P_s_—separator pressure (bar).

According to Equation (8), the calculated mass flow rate from the separator at an initial pressure of 70 bar is 6.26 × 10^−3^ kg/s, which is 11.9 times higher than the mass flow rate at a separator pressure of 10 bar (5.24 × 10^−4^ kg/s). Under these conditions, CO_2_ with a high mass flow rate acts as a carrier gas for volatile EO compounds, entraining and removing them from the separator.

As a result, the combined effect of enhanced solvent power and increased CO_2_ mass flow rate leads to significant losses of spearmint EO. Therefore, a separator pressure of 10 bar was selected as optimal for further research.

Changes in separator pressure also influence the composition of spearmint EO. An increase in pressure enhances the solubility of volatile EO compounds, promoting their transfer to the second separator and subsequent entrainment into the atmosphere. In contrast, under the same pressure increase, the relative content of higher-molecular-weight compounds in the separator increases.

For example, the content of the primary spearmint EO component, carvone, changed only slightly when the separator pressure increased from 10 to 50 bar; however, a further increase in pressure caused a sharp decrease in its content, from 56.08% to 10.13%. Similar behavior was observed for borneol, caryophyllene, and germacrene. In contrast, an inverse trend was found for triacontane, a paraffinic compound. Increasing the separator pressure from 10 to 50 bar had little effect on its content, whereas a further pressure increase led to a substantial rise in its proportion, from 2.59% to 65.71% (Table 2). A similar dependence was observed for octacosane and eicosane.

#### 3.8.2. Flow Rate Research

The CO_2_ flow rate is a critical parameter in studying EO solubility in supercritical CO_2_ (SC-CO_2_). The absence of a maximum EO concentration in the extractor indicates that SC-CO_2_ is not saturated with the essential oil under the studied conditions [60].

**Figure 3:**
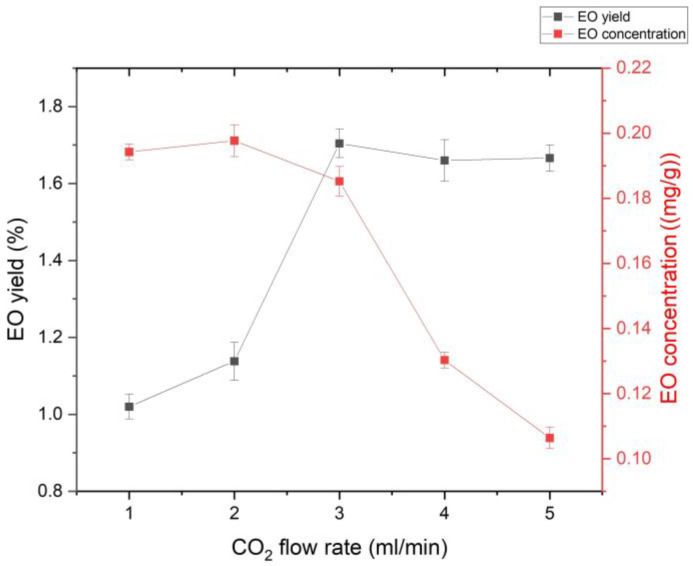
Effect of CO_2_ flow rate on EO yield and concentration.

In this study, the EO concentration showed only minor variation at CO_2_ flow rates between 1 and 3 mL/min (0.194 ± 0.022 mg/g). With a further increase in the CO_2_ flow rate, the EO concentration decreased to 0.106 ± 0.003 mg/g (Figure 3). This decline can be explained by the increase in the total amount of CO_2_ passing through the extractor at higher flow rates, which dilutes the extracted EO. At a CO_2_ flow rate of 3 mL/min, a stable (equilibrium) spearmint EO yield of 0.70 ± 0.04% was observed, indicating saturation of SC-CO_2_ with EO and attainment of the maximum EO concentration (Figure 3). Therefore, a CO_2_ flow rate of 3 mL/min was selected to investigate the effect of CO_2_ density on EO solubility. Under these conditions, SC-CO_2_ is fully saturated with spearmint EO, resulting in the maximum yield and concentration. Similar behavior was reported in [27], where increasing the CO_2_ flow rate from 1.17 to 2.12 g/min led to an increase in red palm oil concentration; no change was observed between 2.12 and 4.57 g/min, and a further increase caused a decline in concentration. Likewise, in [61], changing the CO_2_ flow rate from 2 to 4 mL/min did not affect hemp oil yield, indicating saturation of CO_2_ with hemp oil.

#### 3.8.3. Solubility Research

**Figure 4:**
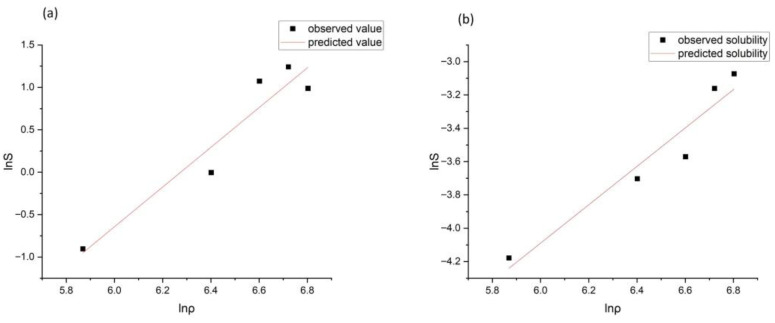
Experimental and predicted values of spearmint EO (**a**) and carvone (**b**) solubility in supercritical CO_2_.

Another important factor influencing EO solubility is the density of CO_2_. As shown in Figure 4a, EO solubility varies markedly with changes in CO_2_ density. In general, EO solubility increased with increasing CO_2_ density; however, a further increase in density from 830.09 to 900.30 kg/m^3^ resulted in a decrease in EO solubility. This behavior may be attributed to the precipitation of high-molecular-weight compounds, such as waxes, in the regulator. At higher CO_2_ densities, the extraction yield of high-molecular-weight compounds, including lutein and β-carotene, from *Mentha spicata* L. leaves has also been reported to increase [19].

Similar trends have been observed in previous studies. In [62], the maximum spearmint EO yield (2.03%) was obtained at 45 °C and 90 bar. In [20], increasing the extractor pressure from 100 to 200 bar led to an increase in EO yield from 35 to 45 mg/g, whereas a further pressure increase to 300 bar caused a decline in yield to 40 mg/g. Likewise, in [63], the EO yield of *Mentha spicata* L. leaves at 45 °C was reported to be 0.065%, 0.265%, and 0.295% at pressures of 85, 100, and 120 bar, respectively.

Carvone solubility followed a trend similar to that of total spearmint EO solubility in supercritical CO_2_. As shown in Figure 4b, carvone solubility increased with increasing CO_2_ density.

The statistical parameters SSE, RMSE, and R^2^ for EO and carvone solubility confirm the adequacy of the selected model (Table 3).

The absolute term of the Chrastil equation, a_0_ (Equation (7)), is a constant that depends on the molecular mass and melting temperature of both the dissolved substance and the solvent (SC-CO_2_). The Chrastil model assumes specific interactions between the solute and the solvent, leading to the formation of solvation complexes. The constant a_1_ represents the average number of solvent molecules forming a solvation complex with the dissolved substance. In addition, the main components of essential oils are terpenoids, which exhibit high solubility in SC-CO_2_. For example, monoterpenes are widely used in oxidation reactions with molecular oxygen in an SC-CO_2_ environment, where CO_2_ acts as the solvent [64].

The value of the a_1_ constant may indicate that fewer solvent molecules are required for the formation of a solvation complex (Table 3). For example, in [65], the a_1_ constant of the Chrastil equation was reported as 1.486. Lavender (*Lavandula angustifolia* L.) essential oil, which also consists predominantly of terpenoids with high solubility in the solvent, exhibited an EO solubility of 28.8 g/kg at a CO_2_ density of ρCO_2_ = 788.92 kg/m^3^, whereas in the present study, the solubility reached 35.5 g/kg.

In contrast, a much higher a_1_ value (7.10) was reported for vitamin E. At CO_2_ densities of ρCO_2_ = 784.29–787.97 kg/m^3^, vitamin E solubility ranged from 3.79 to 4.51 × 10^−5^ mg/kg [29]. Furthermore, the absolute value of the a_0_ constant tends to be minimal for low-molecular-weight compounds and increases for high-molecular-weight compounds. For vitamin E, the a_0_ value was reported as 51.93, whereas in the present study it ranged from 2.2 to 14.7.

Similarly, in a study of argan oil (*Argania spinosa* L.) solubility—comprising mainly mono- and polyunsaturated fatty acids, carotenoids, sterols, and other high-molecular-weight compounds—the absolute value of the free constant was 39.72 [66]. Comparable results were obtained in [19], where the solubility of trans-β-carotene, trans-lutein, and cis-lutein in SC-CO_2_ was investigated. The corresponding absolute values of the a_0_ constant were 34.27, 42.76, and 30.80, respectively, while the average numbers of solvent molecules forming the solvation complex (a_1_) were 5.67, 10.02, and 9.79, respectively.

Overall, the Chrastil solubility model, which is based on the relationship between solubility and solvent density, provides high accuracy in describing the solubility of both total EO and carvone in supercritical CO_2_.

The values of total phenolic content (TPC) of spearmint EO obtained at different CO_2_ densities are presented in Table 4. At low CO_2_ density, lower TPC values were observed. In particular, at a CO_2_ density of 353.91 kg/m^3^, the TPC was 72.3 ± 2.2 mg EGA/g, whereas at the maximum CO_2_ density, it decreased to 24.1 ± 0.7 mg EGA/g. An increase in CO_2_ density generally enhances its solvent power, leading to the extraction of high-molecular-weight compounds such as waxes and lipids. As a result, although the EO yield increases at higher CO_2_ densities, the TPC decreases due to dilution of phenolic compounds by co-extracted high-molecular-weight components. 

Antioxidant activity often correlates with total flavonoid content and TPC. This relationship was demonstrated in [67], where the maximum antioxidant activity of degreased rice bran extract obtained using 80% methanol was 240.23 ± 2.12 ppm, and the highest TPC (3.67 ± 0.45 mg EGA/g) was also observed for the same extract. In comparison, the TPC of *Mentha piperita* L. peppermint EO obtained by SC-CO_2_ extraction at 100 bar and 45 °C from different regions of Pakistan ranged from 31.21 ± 0.96 to 38.19 ± 1.55 g EGA/100 g [68]. In another study, the TPC of an ethanol spearmint extract was reported as 0.75 ± 0.03 mg phenol /100 g [69].

## 4. Conclusions

In the present study, sample preparation methods for *Mentha spicata* L. leaves were investigated. The moisture-absorbing capacity of calcium chloride enabled a reduction in vacuum-drying time by 21.1% and process cost by 26.5%. In addition, the EO content of spearmint leaves after vacuum drying with a desiccant was 0.90 ± 0.22%, compared with 0.83 ± 0.12% for vacuum drying without a desiccant.

An increase in separator pressure induced a higher CO_2_ flow rate, resulting in incomplete separation of EO within the separator. Elevated pressure also enhanced the solubility of volatile components, further contributing to EO losses. It was established that at a separator pressure of 70 bar the EO composition was dominated by high-molecular-weight compounds, whereas at 10 bar the proportion of low-molecular-weight (volatile) components was significantly higher. The EO yield at 10 bar was 1.52 ± 0.05%, compared with only 0.12 ± 0.06% at 70 bar.

The attainment of a maximum EO concentration indicated an equilibrium state, allowing for a more accurate determination of EO solubility. Therefore, solubility determination under dynamic supercritical CO_2_ extraction conditions was shown to be both feasible and practical. The solubility of EO at different CO_2_ densities correlated with the solubility of its main component, carvone. The coefficients of determination (R^2^) for the Chrastil model were 0.9688 for total EO solubility and 0.9275 for carvone solubility.

Furthermore, the total phenolic content decreased with increasing CO_2_ density due to the co-extraction of a larger proportion of high-molecular-weight compounds, which are not phenolic in nature.

Overall, the results obtained in this study may be useful for scaling up essential oil extraction processes and for improving the understanding of essential oil behavior during supercritical CO_2_ extraction. In particular, the demonstrated influence of sample pretreatment, separator pressure, and CO_2_ density provides practical guidelines for optimizing extraction efficiency, selectivity, and product quality. The findings also highlight the importance of controlling phase equilibrium and solubility under dynamic extraction conditions, which is essential for the rational design and industrial implementation of supercritical CO_2_ extraction of aromatic and bioactive compounds from plant materials.

## Figures and Tables

**Table 1:** Spearmint EO content in fresh and dried leaves by different methods.

No.	Drying Methods	Spearmint EO Content in mL/100 g Dried Sample
1	Vacuum drying Т = 30 °C	1.3 ± 0.13 ^a^
2	Convective drying Т = 30 °C	1.2 ± 0.068 ^a,b^
3	Shade	1.1 ± 0.21 ^a,b,c^
4	Convective drying Т = 40 °C	0.98 ± 0.089 ^b,c^
5	Vacuum drying Т = 40 °C with desiccant	0.90 ± 0.22 ^b,c,d^
6	Vacuum drying Т = 40 °C without desiccant	0.83 ± 0.12 ^c,d^
7	Fresh leaves	0.62 ± 0.024 ^d^ *
8	Vacuum drying Т = 50 °C	0.63 ± 0.071 ^d^
9	Convective drying Т = 50 °C	0.27 ± 0.13 ^e^

* EO content is given per 100 g fresh raw material. Different superscript letters within the column indicate significant differences between means according to Tukey’s HSD test (*p* < 0.05).

**Table 2:** Chemical composition of spearmint EO, determined by GC-MS, obtained at different separator pressures.

No.	Name of Compound	Relative Concentration, %						
		10 Bar	20 Bar	30 Bar	40 Bar	50 Bar	60 Bar	70 Bar
**1**	D-Limonene	0.53	0.88	1.01	n/d	2.24	n/d	n/d
2	endo-Borneol	0.44	0.45	0.43	0.44	0.44	n/d	n/d
3	Neodihydrocarveol	0.77	0.86	0.81	0.80	083	n/d	n/d
4	trans-Carveol	0.46	0.57	0.51	0.56	0.50	n/d	n/d
5	Carveol	0.27	0.31	0.28	0.33	0.28	n/d	n/d
6	D-Carvone	54.14	55.08	49.37	46.14	56.08	36.58	10.13
7	Copaene	0.68	0.80	0.82	0.77	0.90	n/d	6.07
8	(-)-.beta.-Bourbonene	2.81	3.22	3.07	3.39	3.78	2.58	n/d
9	Caryophyllene	3.67	3.85	3.57	4.18	3.88	n/d	n/d
10	cis-Muurola-4(15).5-diene	1.18	1.11	1.24	1.46	1.21	1.05	n/d
11	(E)-.beta.-Famesene	0.99	0.90	1.01	1.10	0.86	n/d	n/d
12	(+)-epi-Bicyclosesquiphellandrene	1.91	1.78	1.97	2.47	2.08	1.07	n/d
13	(1R.2S.6S.7S.8S)-8-Isopropyl-1-methyl-3-methylenetricyclo decane	3.74	3.22	3.61	5.01	3.96	2.91	n/d
14	Germacrene D	0.65	0.39	0.48	0.79	0.64	n/d	n/d
15	4-isopropyl-1.6-dimethyl-1.2.3.4-tetrahydronaphthalene	1.45	1.31	1.44	1.90	1.55	1.42	n/d
16	Caryophyllene oxide	0.93	0.74	0.79	0.94	0.96	0.86	n/d
17	4a(2H)-Naphthalenol	0.88	0.96	0.90	1.08	0.98	1.38	n/d
18	.tau.-Muurolol	0.68	0.71	0.72	0.99	0.66	0.76	n/d
19	Eudesma-4(15).7-dien-1.beta.-ol	1.14	1.32	1.24	2.06	1.27	1.10	n/d
20	Hexadecanoic acid. ethyl ester	n/d	n/d	n/d	2.35	2.82	n/d	n/d
21	n-Hexadecanoic acid	2.70	3.63	3.70	2.96	0.20	1.11	n/d
22	1.2-Benzenedicarboxylic acid. bis(2-methylpropyl) ester	1.20	0.78	1.76	2.94	n/d	n/d	n/d
23	Phytol	2.16	2.70	3.09	4.15	2.58	2.57	n/d
24	9.12.15-Octadecatrienoic acid. methyl ester	0.88	0.88	0.86	1.19	0.71	n/d	n/d
25	Octadecanoic acid. ethyl ester	0.48	0.49	0.59	0.72	0.37	n/d	n/d
26	Linoleic acid ethyl ester	1.12	1.04	1.14	1.59	0.97	0.88	n/d
27	9.12-Octadecadienoic acid (Z.Z)-	0.47	0.69	0.72	1.47	0.31	1.22	n/d
28	9.12.15-Octadecatrienoic acid. ethyl ester	3.00	2.77	3.00	3.34	2.66	n/d	n/d
29	9.12.15-Octadecatrienoic acid	1.03	1.63	1.52	2.63	0.91	n/d	n/d
30	Decanedioic acid. dibutyl ester	1.15	0.44	1.52	0.59	n/d	n/d	n/d
31	Eicosane	0.85	1.14	1.24	0.58	1.06	3.84	6.51
32	Hexanedioic acid. bis(2-ethylhexyl) ester	1.51	0.32	1.38	0.30	n/d	2.50	n/d
33	7-Methyl-2-phenylquinoline	2.97	0.43	0.57	n/d	n/d	n/d	6.49
34	Nonacosane	n/d	n/d	0.25	n/d	0.17	1.47	n/d
35	Squalene	0.31	0.33	0.40	0.49	0.45	n/d	n/d
36	Octacosane	0.26	0.39	0.51	n/d	0.33	3.50	5.09
37	Triacontane	2.59	3.89	4.49	n/d	3.36	33.22	65.71
Total		100.00	100.00	100.00	100.00	100.00	100.00	100.00

**Table 3:** Constants and statistical parameters of the Chrastil solubility model.

	Constants			Statistical Parameters		
	а_0_	а_1_	а_2_	SSE	RMSE	R^2^
EO carvone	−14.6962 −11.0021	2.3422 1.1521	−0.0351 −0.0351	0.2766 0.0578	0.2352 0.1075	0.9688 0.9275

**Table 4:** Total phenol content (TPC) values in spearmint EO obtained at various CO_2_ densities.

Density CO_2_, kg/m^3^	353.91	602.58	736.10	830.09	900.30
TFC, mg EGA/g	72.3 ± 2.2	32.2 ± 1.5	22.4 ± 0.1	21.8 ± 0.8	24.1 ± 0.7

## Data Availability

The original contributions presented in this study are included in the article/Supplementary Material. Further inquiries can be directed to the corresponding author.

## Supplementary Materials

The following supporting information can be downloaded at https://www.mdpi.com/article/10.3390/foods15020213/s1: Figure S1: Laboratory unit and scheme for supercritical fluid CO_2_ extraction (LLP Supergidrofobnyie pokrytiya, Russia); Figure S2: Spearmint leaf moisture ratio’s dependence on drying time under vacuum, convection and natural conditions (room temperature 25 ± 3 °C); Table S1: Macro- and micronutrient content in spearmint leaves; Table S2: Coefficients and statistical measures of mathematical models for vacuum drying with the CaCl_2_ desiccant; Table S3: Coefficients and statistical measures of mathematical models for vacuum drying without the CaCl_2_ desiccant; Table S4: Chemical composition of spearmint EO, determined by GC-MS, obtained in different drying conditions.

## Abbreviations

EO	Essential oil
FEMA	Flavor Extract Manufacturers Association
GAE	Gallic acid equivalent
GC-MS	Gas chromatography with mass spectrometer
GOST	Government standard
HM	Heavy metal
JSC	Joint-stock company
LLP	Limited liability partnership
MC	Moisture content
MPC	Maximum-permissible concentration
MR	Moisture ratio
NIST	National Institute of Standards and Technology
RMSE	Root-mean-square error
SC-CO_2_	Supercritical CO_2_
SSE	Sum squared error
TPC	Total phenolic acid
VD	Vacuum drying
