# Supplementary material for: Supercritical Fluid CO2 Extraction of Essential Oil from Spearmint Leaves Dried by Vacuum Drying with a Desiccant
Source: Foods. 2026 Jan 7;15(2):213. doi: 10.3390/foods15020213 (PMC12840100; doi:10.3390/foods15020213)
Supplement: Supplementary file 1 [file foods-15-00213-s001.zip › foods-4010073-supplementary.pdf]

Article

Supercritical Fluid CO_2 Extraction of Essential Oil from Spearmint Leaves Dried by Vacuum Drying with a Desiccant

Rustam Tokpayev [1], Zair Ibraimov [1,*], Khavaza Tamina [1], Bauyrzhan Bukenov [1], Bagashar Zhaksybay [1], Amina Abdullanova [1], Yekaterina Chshendrygina [1], Kanagat Kishibayev [1] and Luca Fiori [2]

[1] Center of Physical and Chemical Methods of Research and Analysis, al-Farabi Kazakh National University, 96A, Tole bi, Almaty 050012, Kazakhstan
[2] Department of Civil Environmental and Mechanical Engineering, University of Trento, via Mesiano 77, 38123 Trento, Italy
* Correspondence: zair.ibraimov@kaznu.edu.kz

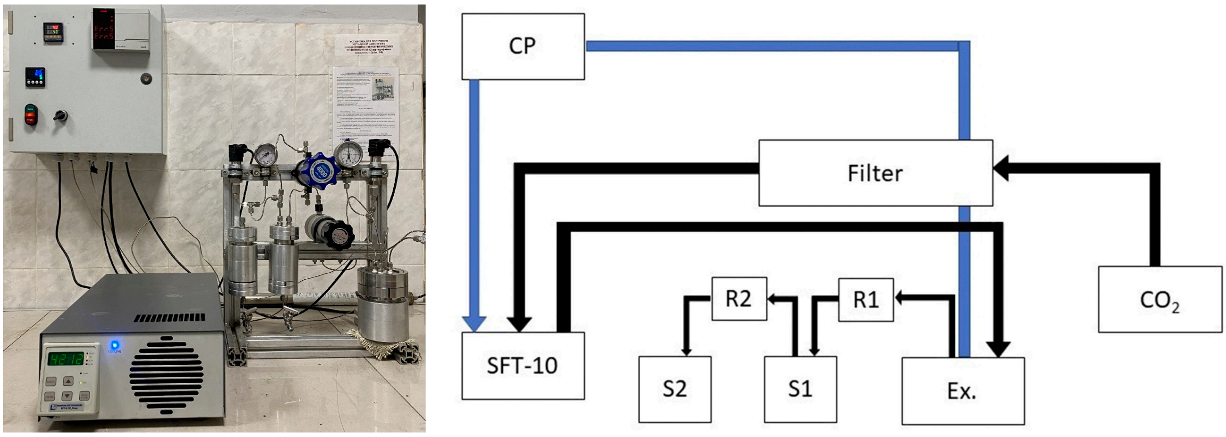

Legend: CP – control panel; SFT-10 – carbon dioxide pump; Filter - filter element; S1, S2 – separator 1, 2; R1, R2 – pressure regulator; Ex – extractor; CO_2 – carbon dioxide cylinder

Figure S1. Laboratory unit and scheme for supercritical fluid CO_2 extraction (LLP Supergidrofobnyie pokrytiya, Russia)

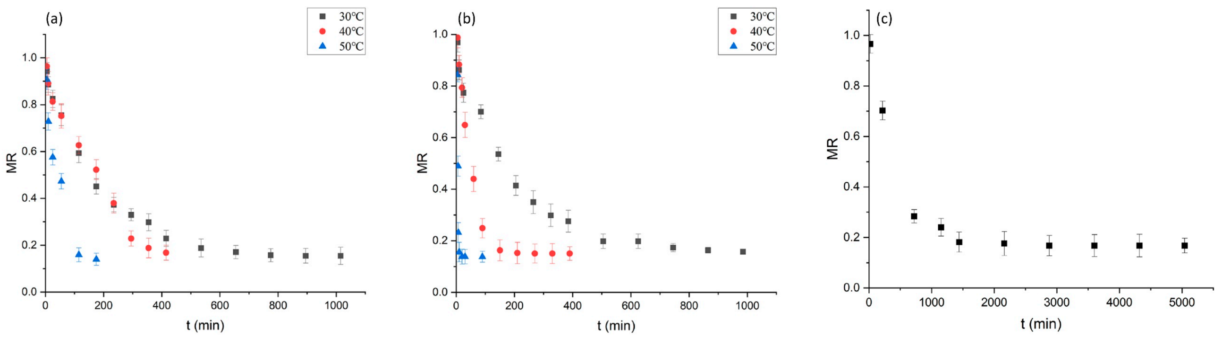

Figure S2. Spearmint leaves moisture ratio dependence on vacuum, convective and natural conditions drying time (room temperature 25±3 °C)

Table S1. Macro- and micronutrients content in spearmint leaves

Nutrients	Nutrients content, mg/g	MPC in soil, mg/g
**macronutrients**		
K	49500±400	-
Mg	4550±30	-
Ca	6150±340	-
Na	3270±30	-
Sum micronutrients	63480±810	
Fe	570±30	-
Mn	100±1	1500
Cu	21±0	55
Zn	70±1	100
Pb	5±0	30
Cd	2±0	-
Ni	7±0	
Sum macronutrients	780±40	
TOTAL:	64260±850	

Table S2. Coefficients and statistical measures of mathematical models for vacuum drying with the $CaCl_2$ desiccant

No.	Equations	**Constants and coefficients**									**SSE**	**RMSE**	**R^2**
		k	n	a	c	k_0	b	k_1	g	h			
**1**	$MR=\exp(-kt)$	0.0020									0.1685	0.1238	0.9766
**2**	$MR=\exp(-kt^n)$	0.0009	1.1205								0.1381	0.1120	0.9808
**3**	$MR=\exp((-kt)^n)$	0.0020	1								0.1685	0.1238	0.9766
**4**	$MR=a\exp(-kt)$	0.0022		1.115							0.0994	0.0951	0.9862
**5**	$MR=\exp(-kt)+c$	0.0024			0.0408						0.1427	0.1139	0.9802
**6**	$MR=a\exp(-k_0t)+b\exp(-k_1t)$			1.153		0.0022	-0.0730	0.0148			0.0965	0.0937	0.9866
**7**	$MR=a\exp(-kt^n)+bt$	0.0491	0.0491	1.151							0.0810	0.0858	0.9887
**8**	$MR=a\exp(-kt)+b\exp(-gt)+c\exp(-ht)$	0.0024		0.5737	0.5737		0.0363		7.271	0.0024	0.0810	0.0858	0.9887
**9**	$MR=a\exp(-kt)+(1-a)\exp(-kat)$	0.0025		1.6730							0.1411	0.1133	0.9804
**10**	$MR=a\exp(-kt)+(1-a)\exp(-kbt)$	0.0020		1			1				0.1685	0.1238	0.9766
**11**	$MR=a\exp(-kt)+(1-a)\exp(-gt)$	0.0022		1.1145					21.05		0.0994	0.0951	0.9862
**12**	$MR=(a+b)/(1+e^{k(t-c)})$	0.0022		0.0176	-2192		127.9				0.0995	0.0951	0.9862

1 - Newton Model, 2 - Page Model, 3 - Modified Page Model, 4 - Handerson and Pabis Model, 5 - Logarithmic Model, 6 - Two-term Model, 7 - Midilli and Kucuk Model, 8 - Mod. Henderson and Pabis Model, 9 - Two-term exponential Model, 10 - Diffusion approach, 11 - Verma Model, 12 - Sigmoid Model

Table S3. Coefficients and statistical measures of mathematical models for vacuum drying without the $CaCl_2$ desiccant

No.	Equations	**Constants and coefficients**									**SSE**	**RMSE**	**R^2**
		k	n	a	c	k_0	b	k_1	g	h			
**1**	MR=exp(-kt)	0.0015									0.1685	0.1238	0.9766
**2**	MR=exp(-kt^n)	0.0006	1.219								0.1696	0.1189	0.9756
**3**	MR=exp((-kt)^n)	0.0015	1								0.1381	0.1073	0.0904
**4**	MR=a exp(-kt)	0.0017		1.113							0.1696	0.1189	0.9756
**5**	MR=exp(-kt)+c	0.0019			0.0546						0.0982	0.0904	0.9859
**6**	MR=a exp(-k_0t)+ b exp(-k_1t)			1.155		0.0017	0.0771	0.0116			0.133	0.1054	0.9808
**7**	MR=a exp(-kt^n)+bt	0.0430	0.0430	1.143							0.0947	0.0889	0.9864
**8**	MR=a exp(-kt)+ b exp(-gt)+c exp(-ht)	0.0018		0.5707	0.5707		0.0334		7.257	0.0018	0.0821	0.0827	0.9882
**9**	MR=a exp(-kt)+(1-a) exp(-kat)	0.0019		1.694							0.0820	0.0827	0.9882
**10**	MR=a exp(-kt)+(1-a) exp(-kbt)	0.0015		1			1				0.1412	0.1085	0.9797
**11**	MR=a exp(-kt)+(1-a) exp(-gt)	0.0017		1.112					21.05		0.1696	0.1189	0.9756
**12**	MR=(a+b)/(1+e$^{k(t-c)}$)	0.0022		0.0176	-2192		127.9				0.0982	0.0904	0.9859

1 - Newton Model. 2 - Page Model. 3 - Modified Page Model. 4 - Handerson and Pabis Model. 5 - Logarithmic Model. 6 - Two-term Model. 7 - Midilli and Kucuk Model. 8 - Mod. Henderson and Pabis Model. 9 - Two-term exponential Model. 10 - Diffusion approach. 11 - Verma Model. 12 - Sigmoid Model

Table S4. Chemical composition of spearmint EO, determined by GC-MS, obtained at different drying conditions

No.	Name of compound	Relative concentration, %								
		Fresh	Vac. 30	Vac. 40	Vac'. 40	Vac. 50	Con. 30	Con. 40	Con. 50	Natural
1	Pinene	n/d	0.12	0.94	0.85	0.37	3.86	2.53	n/d	3.04
2	Myrcene	n/d	0.33	n/d	0.30	1.86	n/d	n/d	n/d	n/d
3	D-Limonene	3.72	2.39	15.00	6.74	8.46	13.76	12.83	7.21	7.16
4	Eucalyptol	2.97	1.53	2.92	2.55	1.63	2.47	2.13	2.52	2.43
5	Ocimene	0.42	0.33	0.48	0.38	0.28	0.35	0.40	n/d	0.40
6	Linalool	n/d	0.59	0.5	0.61	0.80	0.52	0.58	n/d	0.58
7	Borneol	0.39	0.40	n/d	0.38	0.52	0.46	0.47	n/d	0.53
8	Carveol	2.03	0.94	1.75	0.61	1.08	0.24	1.17	1.69	0.86
9	Carvone	77.61	55.42	51.7	52.93	41.42	39.00	42.43	69.57	43.11
10	Bourbonene	0.59	3.57	2.48	2.76	3.31	2.41	3.10	0.98	2.96
11	Gurjenene	n/d	0.75	n/d	0.63	0.80	0.54	0.53	n/d	0.53
12	Copaene	n/d	0.68	n/d	0.30	1.61	0.32	0.48	n/d	0.77
13	Caryophyllene	0.71	4.15	3.75	3.62	4.65	3.56	3.56	1.56	3.34
14	Farnesene	n/d	1.67	1.48	1.46	1.99	1.68	1.65	n/d	1.64
15	Germacrene D	2.59	10.12	n/d	8.98	10.36	9.50	8.86	3.61	8.50
16	Elemene	n/d	1.00	n/d	1.00	0.39	n/d	n/d	n/d	1.11
17	Bicyclogermacrene	n/d	n/d	n/d	n/d	1.30	1.49	1.26	n/d	1.08
18	Cadinene	n/d	n/d	n/d	0.44	1.85	1.34	0.62	n/d	n/d
19	Humulene	n/d	0.89	0.66	0.64	1.10	0.88	0.78	n/d	0.81
20	epi-Bicyclosesquiphellandrene	n/d	4.28	n/d	0.78	1.46	1.94	2.09	n/d	1.89
21	Muurolene	n/d	0.49	n/d	0.49	1.36	n/d	0.90	n/d	0.32
22	Cubebene	n/d	1.64	7.45	1.64	n/d	n/d	1.05	n/d	n/d
23	Total	91.02	91.26	89.09	88.05	86.55	84.30	87.38	87.17	81.02

Fresh – fresh spearmint leaves, Vac 30, Vac 40, Vak 40′, Vak 50– VD at 30, 40°C with/without desiccant, 50; Con 30, Con 40, Con 50 convective drying at 30, 40, 50°C; Natural – natural drying in the shade. n/d – not detected
